# Comprehensive Oncogenic Features of Coronavirus Receptors in Glioblastoma Multiforme

**DOI:** 10.3389/fimmu.2022.840785

**Published:** 2022-04-06

**Authors:** Anjing Chen, Wenguo Zhao, Xiaolong Li, Guangyu Sun, Zhaoyin Ma, Lingyu Peng, Zhongyang Shi, Xingang Li, Jie Yan

**Affiliations:** ^1^ Department of Neurosurgery, Qilu Hospital, School of Medicine, Cheeloo College of Medicine and Institute of Brain and Brain-Inspired Science, Shandong University, Jinan, China; ^2^ Shandong Key Laboratory of Brain Function Remodeling and Jinan Microecological Biomedicine Shandong Labotatory, Jinan, China; ^3^ Ragon Institute of Massachusetts General Hospital (MGH), Massachusetts Institute of Technology (MIT) and Harvard, Cambridge, MA, United States; ^4^ Department of Diagnostics, Medical Integration and Practice Center, Cheeloo College of Medicine, Shandong University, Jinan, China

**Keywords:** SARS-CoV-2, GBM, ACE2, ANPEP, ENPEP

## Abstract

The COVID-19 pandemic caused by SARS-CoV-2 infection has placed health systems under excessive pressure and especially elderly people with cancer. Glioblastoma multiforme (GBM) is a malignant brain tumor with an increasing incidence in elderly individuals, and thereby GBM patients are a vulnerable population during the COVID-19 outbreak. Accumulating studies have implied that SARS-CoV-2 might invade the brain directly *via* coronavirus receptors. However, little is known about SARS-CoV-2 infection in the clinical development of GBM. Here, we explored the oncogenic roles of six coronavirus receptors (ACE2, DPP4, ANPEP, AXL, TMPRSS2, and ENPEP) in GBM using bioinformatics and experimental approaches. We found that ANPEP and ENPEP were significantly increased at both the mRNA and protein levels in GBM compared with normal brain tissue. Kaplan–Meier survival curves and Cox regression analysis demonstrated that high expressions of *ANPEP* and *ENPEP* are associated with poor prognosis and survival. Moreover, all receptors are positively correlated with the immune infiltration levels of monocyte. Furthermore, we identified 245 genes between COVID-19 and coronavirus receptors–correlated genes in GBM and performed a thorough analysis of their protein–protein interaction network, functional signaling pathway and molecular process. Our work explores for the first time the association of coronavirus receptors with GBM and suggests ANPEP and ENPEP as potential therapeutic targets of GBM irrespective of COVID-19.

## Introduction

COVID-19 caused by human severe acute respiratory syndrome coronavirus 2 (SARS-CoV-2) is the most serious pneumonia today and thereby is threatening global public health and the economy (1, 2). As of 11 November 2021, 251,584,730 COVID-19 cases and 5,075,809 deaths have been identified in 188 countries and regions (https://coronavirus.jhu.edu/map.html).

COVID-19 with the primary symptoms such as fever, dry cough, diarrhea, and headache, has substantial morbidity and mortality worldwide (3). Although the lung is the major organ of infection, current studies indicate that SARS-CoV-2 might invade the central nervous system (CNS) region directly, resulting in neurological symptoms, such as dizziness, the loss or disruption of smell, taste, muscular coordination, autonomic respiratory control, lethargy, depression and anxiety (4–9). A case series of 214 hospitalized COVID-19 patients showed that 78 patients (38.4%) and more common (45.5%) in patients with severe infection had neurologic manifestations, namely, acute cerebrovascular events and impaired consciousness (5). The autopsy results of COVID-19 patients exhibited hyperemic and edematous brain tissue and the detection of SARS-CoV-2 RNA in cerebrospinal fluid specimens (10). Furthermore, a population-level observation study reported that elderly and cancer patients had increased susceptibility to virus infection (11). A national analysis of COVID-19 demonstrated that 20% of COVID-19 deaths had active cancer (12). A meta-analysis of 15 studies with 3,019 COVID-19-infected cancer patients showed that the overall fatality rate was 22.4% (13). Glioblastoma multiforme (GBM) is identified as a fast-growing and aggressive brain tumor with an increased incidence in the elderly population (14). Therefore, GBM can be considered as the most vulnerable disease during the COVID-19 pandemic (15). In particular, one cohort of 41 diffuse glioma patients infected by SARS-CoV-2 in France showed that 16 patients (39%) died after a median delay of 13 days, which is higher than the general and noncancer population, although the researcher declared that the mortality rate was overestimated and should be taken with caution due to multiple limitations (16).

Traditionally, effective viral entry is the first line of SARS-CoV-2 infection and determines the range of infected organs. It has been well established that the entry of coronaviruses into target tissues requires: 1) the binding of the spike (S) protein of coronaviruses to cellular receptors, which facilitates virus attachment to the cell surface; and 2) the priming of S protein by cellular proteases, which undertakes S protein cleavage to fuse cell membranes (17–19). Accumulating studies have provided bodies of evidence that the following coronavirus receptors play important roles in coronavirus cell entry: angiotensin-converting enzyme 2 (ACE2) (20–24), a type I transmembrane protein, is characterized as a key determinant cellular receptor for SARS-CoV-2 (20–25); TMPRSS2 is identified as a type II transmembrane serine protease and employed for S protein priming of SARS-CoV-2 (26–29); dipeptidyl-peptidase 4 (DPP4), also known as CD26, is a transmembrane glycoprotein and functions as a receptor for the Middle East Respiratory Syndrome coronavirus (MERS-CoV), which is phylogenetically correlated with SARS-CoV-2 (30, 31). Recent studies have demonstrated that the S1 domain of COVID-19 S protein potentially interacts with DPP4 when the virus enter cells of the respiratory tract (32–36); Alanyl aminopeptidase (ANPEP), also named as CD13, is a receptor for human coronavirus-229E (37). A correlation between ANPEP and ACE2 implies that ANPEP is relevant in SARS-CoV-2 cell entry (38, 39); Tyrosine-protein kinase receptor UFO (AXL) specifically interacts with the S protein of SARS-CoV-2 according to tandem affinity purification (TAP)–mass spectrometry analysis, and its overexpression in HEK293T cells promotes viral entry (40); Glutamyl Aminopeptidase (ENPEP), a type II integral membrane protein, is identified as a candidate co-receptor for SARS-CoV-2 based on the co-expression with ACE2, although its involvement in virus infection is not firmly supported (38, 41). To date, higher ACE2 expression in GBM than in GBM-adjacent tissue has been detected in glioma tissues removed from one COVID-19 patient (42). The association of ACE2 and other coronavirus receptors with the pathogenicity of GBM still needs to be explored to better understand how SARS-CoV-2 infection affects the clinical characteristics of GBM patients. In this study, we investigated the expression profiles of six coronavirus receptors in normal brain and GBM tissues by bioinformatics and experimental approaches. We also conducted a correlation analysis between coronavirus expression and prognosis and immune filtration using various web services. Furthermore, to explore the potential molecular mechanism of coronavirus receptors in GBM and COVID-19, the protein–protein interaction network, functional signaling pathway and molecular process regulated by common genes between COVID-19 and coronavirus receptor-correlated genes in GBM were analyzed.

## Material and Methods

### Gene Expression Analysis

The mRNA expression profiles of ACE2 (NP_001358344.1), DPP4 (NP_001926.2), ANPEP (NP_001368853.1), AXL (NP_068713.2), TMPRSS2 (NP_001128571.1), and ENPEP (NP_001968.3) in human and mouse brain were accessed by the Human Protein Atlas database (https://www.proteinatlas.org/). The gene expression values in the glioblastoma cell lines LN018, LN215, LN229, LN319 and BS149 from GDS4468 were downloaded from the Gene Expression Omnibus (GEO) profile (https://www.ncbi.nlm.nih.gov/geoprofiles). The normalized RNA-Seq data in transcripts per million (TPM) of gene expression from The Cancer Genome Atlas (TCGA) datasets based on the clinical features (gender, age, race) of GBM were obtained from an interactive web resource, UALCAN (http://ualcan.path.uab.edu/analysis-prot.html) (normal, n = 5; tumor, n = 156).

### Protein Expression Analysis

The clinical tissue chip of GBM (HBraG090PG01) was purchased from Outdo Biotech Co., Ltd. (Shanghai, China) (normal brain, n = 3; GBM, n = 25). Immunohistochemistry (IHC) was conducted as follows: the sections were dewaxed in xylene, rehydrated in grade alcohol, and then incubated with 5% bovine serum albumin to block nonspecific antigen binding. Afterwards, sections were probed overnight at 4°C with primary antibodies (Abcam, Cambridge, USA) against ACE2 (ab108252, 1:6,400), TMPRSS2 (ab92323; 1:4,000), DPP4 (ab215711; 1:400), AXL (ab219651; 1:500), ANPEP (ab108310; 1:1,600), and ENPEP (ab155991; 1:100) and then incubated with a secondary antibody against rabbit IgG (ZSGB-BIO, Beijing, China) for 120 min at 37°C. The sections were stained with diaminobenzidine and counterstained with hematoxylin. The score of IHC-based protein expression was calculated by Aipathwell (Servicebio) according to the intensity of cytoplasmic staining (no staining = 0; weak staining = 1, moderate staining = 2 and strong staining = 3), and H-Score (H-SCORE = ∑(I × i) = percentage of weak intensity area × 1) + (percentage of moderate intensity area × 2) + (percentage of strong intensity area × 3). In addition, representative immunohistochemistry images of the cerebral cortex of normal brain were extracted from the Human Protein Atlas database.

### Survival Analysis

Kaplan–Meier survival curves and Cox regression analysis were conducted using R language to assess the correlation of receptor expression and clinical outcomes. Patients were divided into high expression and low expression groups based on the median receptor expression levels. The log rank test was used to calculate the significance of survival differences caused by receptors expression. Univariate and multivariate Cox analyses were used to assess the expression of receptors and clinical characteristics of GBM patients using the CGGA (Chinese Glioma Genome Atlas) (http://www.cgga.org.cn/).

### Immune Infiltrate and Subtype Analysis

The functional heatmap table of the association between coronavirus receptors and the infiltration level of monocyte, dendritic cells (DCs), natural killer (NK) cells and eosinophil in GBM (n = 153) was investigated by the “Immune-Gene” module of the Tumor Immune Estimation Resource (TIMER2.0) (http://timer.cistrome.org/) platform, which comprised immune infiltrate data from TCGA patients. The red indicates a significant positive association, the blue indicates a significant negative association, and the gray presents a non-significant result.

### Gene Enrichment Analysis in GBM

The top 100 receptor-correlated targeting genes were extracted from the “similar gene detection” module of Gene Expression Profiling Interactive Analysis (GEPIA2, http://gepia2.cancer-pku.cn/#index) using GBM tumors from the TCGA dataset and normal brain samples from the Genotype-Tissue Expression Project (GTEx) dataset. An intersection analysis was performed by E Venn (http://www.ehbio.com/test/venn) to compare the top 100 similar genes among each coronavirus receptors. The KEGG (Kyoto Encyclopedia of Genes and Genomes) enrichment pathways and GO (Gene Ontology) analyses were conducted as follows: the top 100 similar genes of each receptor were uploaded to the Database for Annotation, Visualization and Integrated Discovery (DAVID; https://david.ncifcrf.gov/) with the settings of the selected identifier (“OFFICAL_GENE_SYMBOL”), species (“*Homo sapiens*”) and functional annotation chart. The enriched pathways with *P*-values <0.05 were finally visualized with bubble chart and network chart by the R language package.

### Co-Expression Analysis of Genes in GBM and COVID-19

The COVID-19 genes were retrieved from the Comparative Toxicogenomics Database (CTD) (https://ctdbase.org/) (The downloaded file is CTD_D000086382_genes_20210411080946) (43). To compare the common genes between COVID-19 and receptor-related genes in GBM, an intersection analysis of COVID-19 genes and top 100 similar genes from each receptor in GBM was conducted by Jvenn, an interactive Venn diagram viewer (https://bioinfogp.cnb.csic.es/tools/venny/index.html). The direct interacting proteins among common genes and six receptors were further determined through the Search Tool with the multiple proteins by Names/Identifiers from the STRING server (https://cn.string-db.org/) and First Neighbors of Selected Nodes from Cytoscape software. The combined score was calculated from STRING with the selected parameters of homolog and experimentally determined interaction. Furthermore, the hierarchical clustering analysis was conducted on both row variables (direct interacting proteins) and column variables (coronavirus receptors) by the library pheatmap function in the R package. In addition, KEGG enrichment pathways and GO functional and molecular processes of common genes were analyzed as described in *Gene Enrichment Analysis in GBM*. The model for the regulation of ANPEP and ENPEP in GBM against coronavirus infections was drawn by BioRender (https://biorender.com/).

### Protein–Protein Docking

The crystallized structure files of the SARS-CoV-2 receptor binding domain (RBD) of S1 subunit of the S protein (isolated from 6M0J), ENPEP (4KX7), and ANPEP (4FYQ) were downloaded from the RCSB Protein Data Bank (PDB) (https://www.rcsb.org/). The possible binding configurations between ligands (SARS-CoV-2 RBD) and the receptor candidates (ANPEP and ENPEP) were searched by the ZDOCK server (https://zdock.umassmed.edu/), a rigid molecular docking approach. The best cluster (Top1 prediction) was selected and then analyzed as follows: the binding free energy (kcal mol^−1^) and a dissociation constant (Kd) were processed using the tools of the PRODIGY web server (https://bianca.science.uu.nl/prodigy). The buried surface area (BSA) (Å2) was shown with the sum of contacting surface values for each protein in the complex using the PDBePISA program (https://www.ebi.ac.uk/msd-srv/prot_int/pistart.html). The graphical images were generated by PyMOL software.

## Results

### Summary of SARS-CoV-2 in the Central Nervous System (CNS) and Cancer

COVID-19 patients have been frequently reported to show neurologic manifestations, namely, headache, dizziness, depression, lethargy, impaired sense of smell and taste, and loss of muscular coordination and autonomic respiratory control (4–9, 44, 45) (**Table 1**). Accumulating evidence from autopsy tissues of COVID-19 patients has revealed that SARS-CoV-2 RNA is detected in brain tissue, cortical neurons, neural and capillary endothelial cells in frontal lobe tissue, olfactory nerve, gyrus rectus and brainstem (46–49). Human brain organoids also exhibit the neuroinvasive capability of SARS-CoV-2 (50, 51). However, how SARS-CoV-2 directly infects the central nervous system (CNS) is still unclear.

**Table 1:** SARS-CoV-2 infection in the central nervous system and cancer.

	COVID-19 in the central nervous system (CNS)	COVID-19 with cancer
Clinical signs	Headache, dizziness, loss or disruption of the sense of smell (anosmia/dysosmia), taste (ageusia/dysgeusia), loss of muscular coordination (ataxia), loss of autonomic respiratory control, lethargy, depression and anxiety (4–9, 44, 45)	Higher prevalence of chest distress, higher death rate, higher rates of ICU admission, higher rates of having at least one severe or critical symptom in COVID-19 patients with lung cancer, gastrointestinal cancer, breast cancer, hematologic cancer, or metastatic cancer (55, 56); Increased virus-associated lymphopenia in cancer patients (57).
Viral detection	SARS-CoV2 is detected in brain tissue (46), cortical neurons (47), neural and capillary endothelial cells in frontal lobe tissue (48), the olfactory nerve, the gyrus rectus and the brainstem in autopsy tissue obtained from COVID-19 patients (49). The neuroinvasive capability of SARS-CoV-2 is also determined in human brain organoids (50, 51).	Prolonged viral shedding and higher viral loads in cancer patients (57, 58).
Receptor expression	ACE2 is expressed in the substantia nigra and brain ventricles, the piriform cortex, neurons and some nonneuron cells (astrocytes and oligodendrocytes from the middle temporal gyrus and posterior cingulate cortex) (52–54); TMPRSS2 is present in oligodendrocyte precursor cells, astrocytes and microglial cells of the neurovascular units (53, 54). DPP4 and ANPEP are distributed in astrocytes and microglial cells of neurovascular units (54).	ACE2 is downregulated in hepatocellular carcinoma (59), non-small cell lung cancer (NSCLC) (60), breast tumors (61), pancreatic ductal adenocarcinoma (62), and gallbladder cancer (63); ACE2 and TMPRSS2 are both upregulated in colorectal tumor (64) and lung cancer (65). TMPRSS2 is downregulated in head and neck cancer (66).

It has been well established that SARS-CoV-2 binds to host cells through its S protein to ACE2 (20–25), and subsequently the arginine and lysine residues of ACE2 are cleaved by TMPRSS2, which is an important step for S protein priming before viral cell entry (26–29). In addition to *ACE2* and *TMPRSS2*, several other molecules have also been suggested to participate in SARS-CoV-2 cell entry, such as *DPP4*, *ANPEP*, *AXL*, and *ENPEP* (30–41). *ACE2* is detected in the CNS, namely, substantia nigra and brain ventricles, piriform cortex, neurons and some nonneuronal cells (astrocytes and oligodendrocytes from the middle temporal gyrus and posterior cingulate cortex) (52–54). TMPRSS2 is observed in oligodendrocyte precursor cells, astrocytes and microglial cells of the neurovascular units (53, 54). DPP4 and ANPEP are distributed in astrocytes and microglial cells of neurovascular units (**Table 1**) (54).

COVID-19 patients with lung cancer, gastrointestinal cancer, breast cancer, hematologic cancer, or metastatic cancer have experienced a higher death rate, ICU admission, and at least one severe or critical symptom (e.g., chest distress) (55, 56). Meanwhile, an increase in virus-associated lymphopenia, prolonged viral shedding and higher viral loads has also been observed in cancer patients (57, 58). Nevertheless, compared with normal tissue, the downregulation of ACE2 has been identified in hepatocellular carcinoma (59), non-small cell lung cancer (NSCLC) (60), breast tumors (61), pancreatic ductal adenocarcinoma (62), and gallbladder cancer (63), while the upregulation of ACE2 and TMPRSS2 has been identified in colorectal tumors (64) and lung cancer (65). TMPRSS2 is decreased in head and neck cancer (66). To date, immunohistochemical staining of glioma tissues surgically removed from one COVID-19 patient showed that ACE2 expression is higher in GBM than in GBM-adjacent tissue (42), but little is known about whether other coronavirus receptors are expressed in GBM (**Table 1**). To understand the pathogenesis and development of SARS-CoV-2 in GBM, we subsequently carried out bioinformatics analysis using various web programs to identify oncogenic features of ACE2, DPP4, ANPEP, AXL, TMPRSS2 and ENPEP in GBM.

### Expression Patterns of Coronavirus Receptors in Normal Brain Tissue/Regions

**Figure 1:**
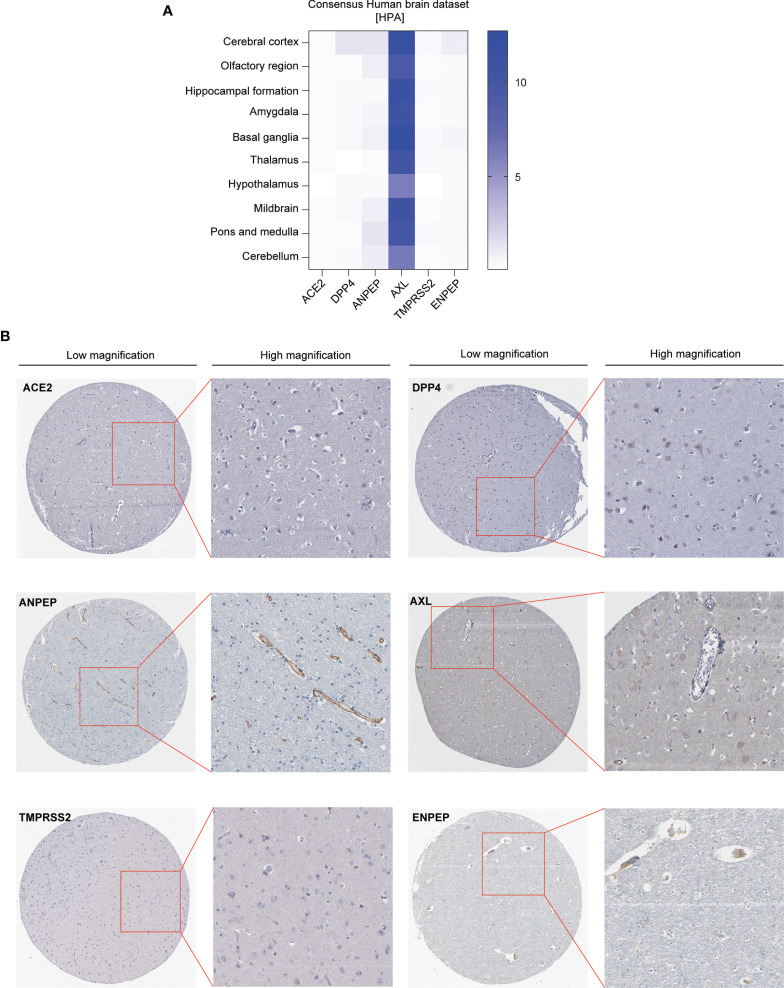
Expression profile of coronavirus receptors in different regions of the brain. **(A)** Human brain datasets. Heatmap of the expression profiles of ACE2, DPP4, ANPEP, AXL, TMPRSS2, and ENPEP extracted from the consensus human brain datasets of the Human Protein Atlas (n = 441) **(B)** Representative image of immunohistochemistry images of coronavirus receptors in the cerebral cortex of human brain (source: The Human Protein Atlas; https://www.proteinatlas.org/humanproteome/brain) (n = 2).

We first analyzed the expression pattern of coronavirus receptors mRNA in different regions of the brain in humans using Human Protein Atlas (HPA) datasets, namely, cerebral cortex, olfactory region, hippocampal formation, amygdala, basal ganglia, thalamus, hypothalamus, midbrain, pons and medulla, and cerebellum. As shown in **Figure 1A**, human *ACE2* was negligibly expressed in all detected regions. Human *DPP4* is detected in the cerebral cortex, with little distribution in other regions. Human *ANPEP* is distributed in some regions, namely, the cerebral cortex, olfactory region, amygdala, midbrain, pons, medulla and cerebellum. Human *AXL* was highly expressed in all detected regions, whereas human *TMPRSS2* was negligibly expressed in the tested regions. Human *ENPEP* is exhibited in many regions, namely, the cerebral cortex, hippocampal formation, basal ganglia, and pons and medulla.

Furthermore, immunohistochemistry analysis of these coronavirus receptors expression in the cerebral cortex was extracted from the HPA database as follows: ACE2 and TMPRSS2 protein are negligible in the cerebral cortex; DPP4 occurs in glial cells and neuronal cells; ANPEP is observed in endothelial cells; AXL and ENPEP are present in endothelial cells and neuronal cells (**Figure 1B**).

### Expression Profiles of Coronavirus Receptors in GBM

**Figure 2:**
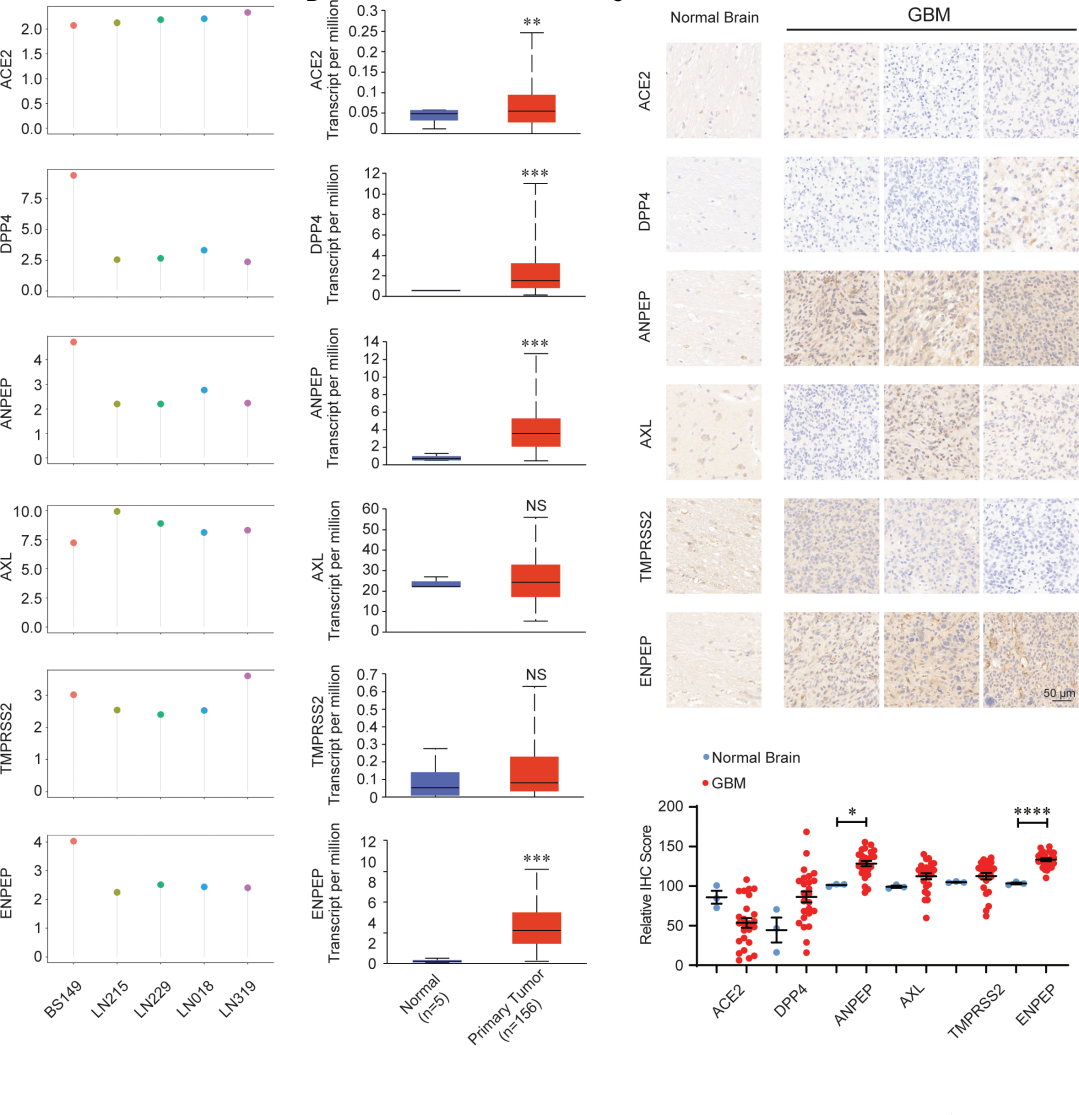
Expression pattern of coronavirus receptors in glioblastoma multiforme (GBM). **(A)** CoV receptor mRNA in glioblastoma cell lines LN018, LN215, LN229, LN319, and BS149 (recurrent glioblastoma) from GDS4468. **(B)** Coronavirus receptor mRNA between normal (n = 5) and GBM (n = 156) tissues extracted from the TCGA database by the UALCAN program. **(C)** Representative images of immunohistochemistry images of receptors in normal (n = 3) and GBM (n = 27) tissue chips. **(D)** Histologic scores. Mean +SEM. Significance comparison is to normal people. Scale bar = 50 μm. *P < 0.05; **P < 0.01; ***P < 0.001; ****P < 0.0001, NS: no significance P > 0.05.

We further analyzed the expression of coronavirus receptors in GBM to dissect their oncogenic role. RNA expression data available on public database were extracted from cell lines, normal and tumor samples. Firstly, GEO profile GDS4468 shows the expression profiles of receptors in following glioblastoma cell lines (LN018, LN215, LN229, LN319 and BS149): human *ACE2*, *AXL*, and *TMPRSS2* are widely expressed in these five cell lines; meanwhile, human *DPP4*, *ANPEP*, and *ENPEP* are highly distributed in BS149, followed by LN018, LN215, LN229, and LN319 (**Figure 2A**). Secondly, we analyzed the expression of coronavirus receptor genes in the GBM dataset using the UALCAN program. *ACE2*, *DPP4*, *ANPEP*, and *ENPEP* were significantly upregulated in GBM patient samples, while *AXL* and *TMPRSS2* were comparable between normal and GBM samples (**Figure 2B**). To validate these observations, we performed an immunohistochemical analysis to identify the expression of coronavirus receptor proteins in pathological GBM tissue chips. As shown in **Figure 2C**, ANPEP and ENPEP protein were markedly increased in GBM patients compared with normal people. Possibly due to the small sample size, no significant differences in ACE2, DPP4, AXL, and TMPRSS2 protein were observed between normal and tumor patients.

Thirdly, we dissected the relationship between the expression of coronavirus receptors and clinical characteristics (**Figure S1**). For age, the levels of *ACE2* and *DPP4* are increased in patients under 60 years old. The levels of *ANPEP* and *ENPEP* were significantly different among patients of different ages and peaked at 60–80 years old. Nevertheless, the levels of *AXL* and *TMPRSS2* were not significantly different in patients of various ages. For gender, the expression levels of all six receptors were not significantly different between male and female. For race, the levels of ACE2, DPP4, TMPRSS2, and ENPEP were comparable among the three races. Nevertheless, the level of AXL was higher in Asians than in Caucasian, while ANPEP was lower in Asian than in Caucasian.

### Survival Analysis of Coronavirus Receptors in GBM

**Figure 3:**
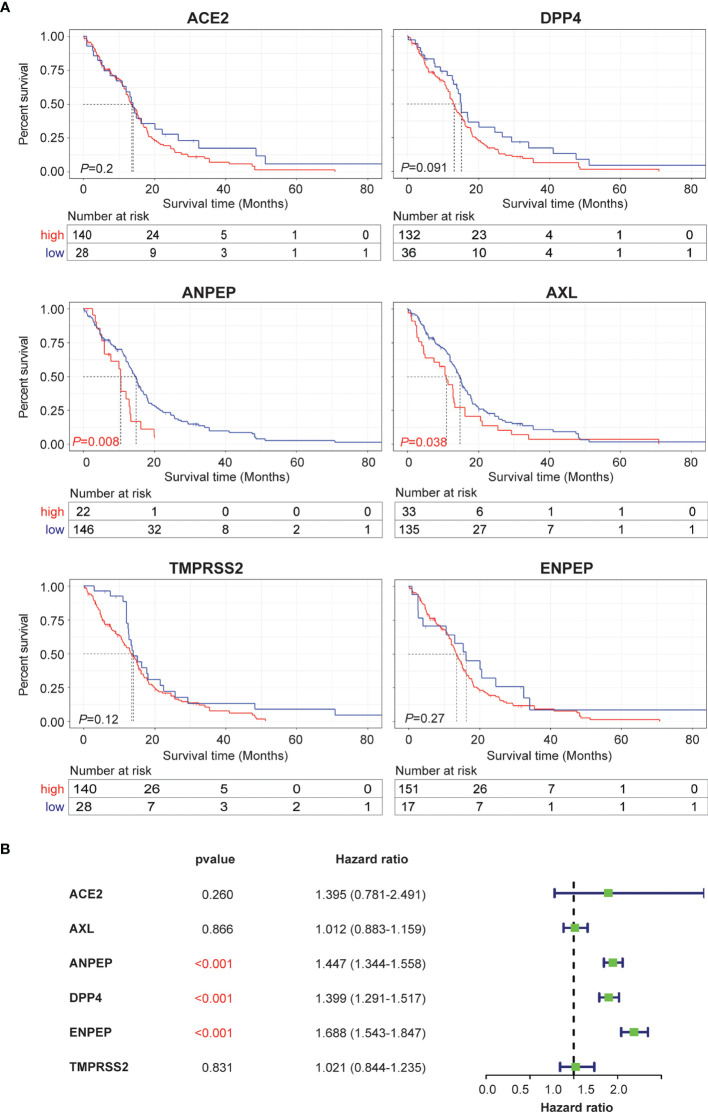
Prognosis and survival analysis of coronavirus receptors in GBM. **(A)** Kaplan–Meier survival curves in the TCGA database. Red indicates high expression, and blue indicates low expression (n = 168). **(B)** Forest plot for the univariate Cox proportional hazard regression model in the CGGA database (n = 216).

To evaluate whether coronavirus receptors expression levels are associated with tumor prognosis in GBM, Kaplan–Meier survival curves were generated with TCGA and CGGA data. As shown in **Figure 3A**, high levels of ANPEP and AXL were significantly linked to poor prognosis for TCGA samples, whereas the correlations of high levels of ANPEP and ENPEP with poor prognosis were identified in CGGA cases (**Figure S2A**). In addition, univariate and multivariate analyses were conducted to evaluate the impact of each coronavirus receptors expression and other clinicopathological factors using the Cox proportional hazard regression model on survival. As shown in **Figure 3B**, the univariate analysis showed that ANPEP (Hazard ratio: 1.447; *P <*0.001), DPP4 (Hazard ratio: 1.399; *P <*0.001), and ENPEP (Hazard ratio: 1.399; *P <*0.001) were negative predictors of survival. Furthermore, multivariate analyses of receptor expression and other clinicopathological variables showed as follows: ENPEP (Hazard ratio:1.243; *P <*0.001), PRS type (Hazard ratio: 1.974; *P <*0.001), grade (Hazard ratio:2.688; *P <*0.001) and age (Hazard ratio: 1.227; *P* = 0.043) were negative predictors of survival; DPP4 (Hazard ratio: 0.883; *P* = 0.043), chemo (Hazard ratio: 0.660; *P <*0.001), IDH_mutation (Hazard ratio: 0.567; *P <*0.001), and 1p19q_codeletion (Hazard ratio: 0.387; *P <*0.001) were positive predictors of survival (**Figure S2B**). Overall, the Kaplan–Meier survival curves indicate that high expression of ANPEP, AXL and ENPEP is correlated with poor prognosis, and the further multivariate analysis demonstrates that high expression of ENPEP can be a negative predictor of survival.

### Association of Coronavirus Receptors With Immune Infiltration in GBM

**Figure 4:**
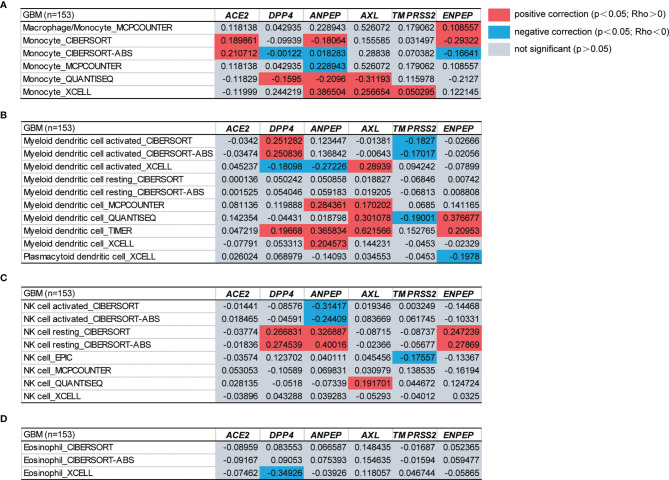
The functional heatmap table of the correlation between coronavirus receptor expressions and immune infiltration levels of different cell types in GBM by TIMER2.0. **(A)** Monocyte. **(B)** DCs. **(C)** NK cell. **(D)** Eosinophil. n = 153. GBM, glioblastoma.

Tumor-infiltrating immune cells are independent predictors of the prominent components of the tumor microenvironment and are closely linked to the initiation, progression or metastasis of cancer (67). We therefore next investigated the relationship between coronavirus receptors and immune infiltration levels across different immune subtypes in GBM. According to the key survival-related immune cells for GBM shown in a gene expression-based study from TCGA datasets, monocytes, DCs, NK cells and eosinophils were selected (68). As shown in **Figures 4A–D**, ACE2 expression was found to be positively correlated with monocyte immune infiltration. DPP4 showed a positive Spearman’s correlation with monocytes, DCs and NK cells in some algorithms, but a negative correlation with eosinophils. The ANPEP expression level was positively correlated with monocytes, DCs and resting NK cells, but negatively correlated with some algorithms of monocytes, activated DCs and NK cells. AXL expression levels were positively correlated with infiltrating levels of monocytes, DCs and NK cells. TMPRSS2 was significantly positively correlated with monocytes, but negatively correlated with DCs and NK cells. Compared with the negative correlation of ENPEP expression levels with in some algorithms of monocytes and DCs, a significant positive association with monocytes, DCs and NK cells was observed. These findings strongly indicated that coronavirus receptors play a vital role in immune infiltration in GBM.

### Enrichment Analysis of Coronavirus Receptor-Related Genes in GBM

**Figure 5:**
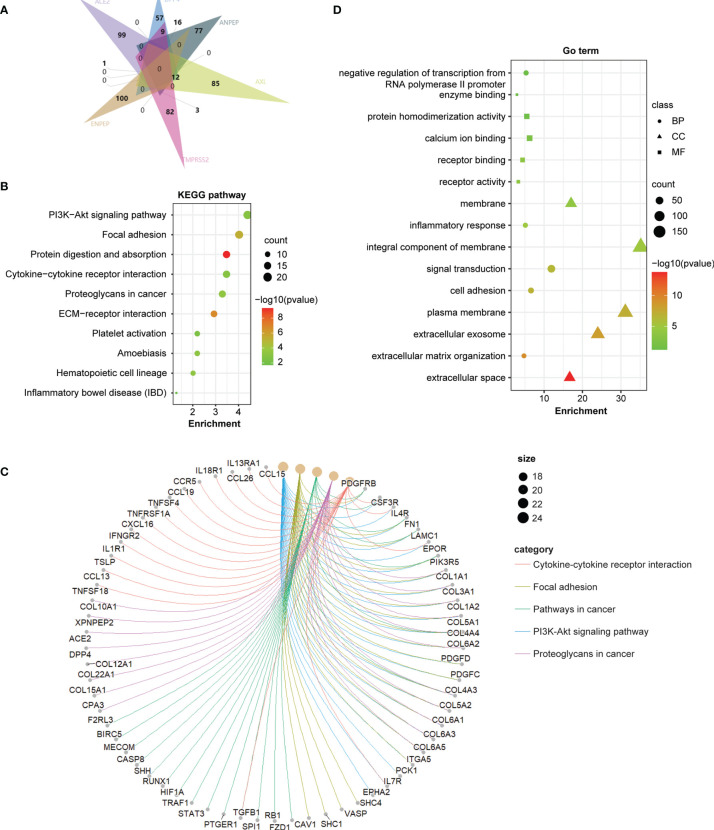
Coronavirus receptor-related gene enrichment analysis in GBM. **(A)** An intersection analysis of the top 100 receptor-correlated genes with GBM among different receptor groups according to the E Venn diagram. **(B)** Bubble chart for KEGG enrichment pathway analysis based on total receptor-related genes. **(C)** The cnetplot of all genes in the yellow module that depicts the linkages of genes and the most important signaling pathways. **(D)** Functional and molecular processes related to coronavirus receptor-related genes in GBM.

To further investigate the molecular mechanism of the coronavirus receptors in GBM tumorigenesis, we obtained the top 100 genes correlated with coronavirus receptors utilizing the combination of GBM tumors from the TCGA dataset and normal brain samples from the GTEx dataset. An intersection analysis among coronavirus receptors revealed the following: 1 common gene between *ACE2* group and *AXL* group (*ZFP36L1*); 9 common genes between *DPP4* and *TMPRSS2*; 12 common genes between *DPP4* and *AXL*; 16 common genes between *DPP4* and *ANPEP*; 3 common genes between *ANPEP* and *TMPRSS2* (**Figure 5A**).

Furthermore, we combined the top 100 related genes from each coronavirus receptor to conduct KEGG and GO enrichment analyses. The enrichment of KEGG pathways revealed that those genes were highly associated with the following pathways during GBM tumor pathogenesis: the PI3K−Akt signaling pathway, focal adhesion, protein digestion and absorption, cytokine–cytokine receptor interaction, proteoglycans in cancer, etc. (**Figure 5B**). The cnetplot displays the relationship of coronavirus receptor-correlated genes in GBM with functional signaling pathways (**Figure 5C**). Furthermore, the GO enrichment analysis of biological process (BP), cellular component (CC) and molecular function (MF) revealed that those coronavirus receptor-correlated genes in GBM are associated with membrane receptor function-related gene terms, namely, signal transduction, integral component of membrane and receptor binding (**Figure 5D**).

### Employment of Coronavirus Receptor-Related Genes in GBM and COVID-19

**Figure 6:**
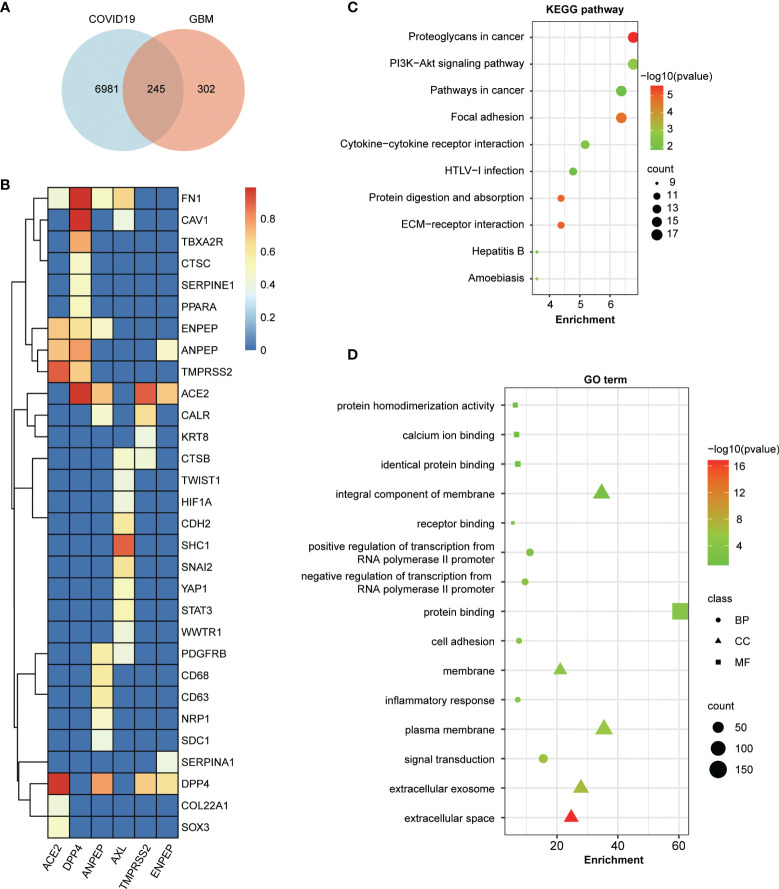
Enrichment analysis of COVID-19-related genes and coronavirus receptor-related genes in GBM. **(A)** An intersection analysis between 7,230 COVID-19 genes downloaded from the Comparative Toxicogenomics Database (CTD) and the top 100 related genes of each receptor in GBM. A total of 245 common genes were identified between COVID-19 and coronavirus receptor-correlated genes in GBM. **(B)** Hierarchical clustering analyses of the direct interaction proteins across 245 common genes with coronavirus receptors. The color key represents the combined score calculated by the selected parameters of the homolog and experimentally determined interaction of the STRING program. Red, yellow and blue refer to high, medium and low combined scores, respectively. **(C)** Bubble chart for KEGG enrichment pathway analyses of common genes. **(D)** Bubble chart for GO functional and molecular processes of common genes.

To address the potential relationship between COVID-19 and coronavirus receptor-associated genes in GBM, an intersection analysis was applied using a Venn diagram. As shown in **Figure 6A**, 245 common genes were found between COVID-19 and coronavirus receptor-correlated genes in GBM. To further dissect the depth of the disease and predict phenotypic–genotypic associations, the direct interacting proteins with coronavirus receptors were identified through STING and Cytoscape software, resulting in 30 genes shown in **Figure 6B**. Coronavirus receptors can interact with each other, such as the binding of ACE2 to TMPRSS2, DPP4, ANPEP or ENPEP; the binding of DPP4 to TMPRSS2, ANPEP or ENPEP; and the binding of ANPEP to ENPEP. AXL can bind to many genes but not coronavirus receptors. FN1 gene can be recognized by four coronavirus receptors, namely, ACE2, DPP4, ANPEP, and AXL. The KEGG pathway enrichment analysis showed that 245 common genes were highly associated with proteoglycans in cancer, the PI3K−Akt signaling pathway, pathways in cancer, focal adhesion, and cytokine–cytokine receptor interactions (**Figure 6C**). Moreover, the GO enrichment analysis showed that 245 common genes were involved in signal transduction-related gene terms, namely, protein binding, integral component of membrane and signal transduction (**Figure 6D**).

## Discussion

### How Does the Virus Enter the Brain?

SARS-CoV-2 infections had neurologic manifestations in 38.4% of patients and 45.5% of severe patients in a case analysis, usually along with headache, dizziness, impaired consciousness, and smell and taste disorders (5, 44, 45). Remarkably, SARS-CoV-2 RNA has been detected in many different brain tissues, namely, cortical neurons, frontal lobe tissue, the olfactory nerve and brainstem (46–49), and cerebrospinal fluid specimens (10). However, how the virus affects the brain is still obscure. Accumulating studies have provided the following hypotheses to explain these viral invasions in the brain: (1) through the olfactory route (69, 70), (2) through retrograde routing from the vagal nerve to the medullary cardiorespiratory center in the brainstem (49), and (3) through hematogenous routing from the blood–brain barrier (BBB) and blood–cerebrospinal fluid barrier (BCSFB) (71). Therefore, tight junctions between adjacent endothelial cells form the basic structure of the BBB, which plays a critical role in limiting virus paracellular trafficking and is therefore thought to be the major route for coronavirus entry into the CNS (71).

Interestingly, ACE2 and TMPRSS2, the two widely accepted receptors in SARS-CoV-2 cell entry, have been reported to be relatively low in endothelial cells of the human brain in two studies (52, 72), which is consistent with the results shown in **Figure 1**. Nevertheless, the coronavirus receptors ANEPE, AXL, and ENPEP were detected in the olfactory region and endothelial cells of human brain (**Figures 1B** and **2C**), indicating that SARS-CoV-2 cell entry in human brain might require these three receptors rather than rely on ACE2 and TMPRSS2.

### The Association of Elevated ANPEP and ENPEP Levels With GBM Progression

A majority of studies focus on the immunosuppressive effect of anti-cancer therapies, which results in the increased susceptibility of cancer patients to COVID-19. However, an increasing number of papers have provided evidence that virus can directly interact with tumors, such as the upregulation of the coronavirus receptor ACE2 (73–76), increased SARS-CoV-2-associated lymphopenia, prolonged viral shedding and higher viral loads (57, 58). Therefore, the mechanisms for the increased susceptibility and severity of cancer to SARS-CoV-2 remain unclear.

Immunohistochemical staining of the GBM tissue chip showed that the protein levels of ANPEP and ENPEP were significantly increased in GBM (**Figure 2C**), although the mRNA levels of *ACE2*, *DPP4*, *ANPEP*, and *ENPEP* were upregulated in GBM according to the UALCAN server (**Figure 2B**). In fact, the BS149 cell line generated from recurrent glioblastoma is more malignant than the other four glioblastoma cell lines (LN018, LN215, LN229, and LN319) and has higher mRNA levels of DPP4, ANPEP, and ENPEP (**Figure 2A**), further corroborating the potential oncogenic roles of ANPEP and ENPEP in GBM. Moreover, the levels of ANPEP and ENPEP were significantly upregulated with increasing age and peaked at 60–80 years old (**Figure S1A**), which agrees with the peak incidence of GBM between 70 and 79 years old (77). Kaplan–Meier survival curves and the Cox regression analysis demonstrated that high expression of ANPEP and ENPEP was associated with poor prognosis and that ENPEP was a negative predictor of survival. Furthermore, the direct interacting proteins with ANPEP and ENPEP were FN1, CALR, PDGFRB, CD68, CD63, NRP1, SDC1, and SERPINA1, all of which are involved in the progression of GBM (**Figure 6B**) (78–85). All of these findings suggest that increasing ANPEP and ENPEP levels are associated with GBM progression.

### The Potential Interactions of SARS-CoV-2 With ANPEP or ENPEP by ZDOCK

The protein and protein interaction network showed that ANPEP, ACE2, DPP4, and ENPEP can form a protein complex (**Figure 6B**), indicating that ANPEP and ENPEP might be directly involved in brain-SARS-CoV-2 communication, similar to ACE2, although no data have firmly supported ANPEP and ENPEP as receptors for SARS-CoV-2. To predict the potential molecular interactions of SARS-CoV-2 with ANPEP or ENPEP, we conducted docking simulations through ZDOCK Server, a rigid body computational docking program. The Top1 prediction was selected for the following analysis (**Supplementary Figure 3**). The complex of SARS-CoV-2 RBD with ENPEP has a △G value of −16.7 kcal mol^−1^, Kd value of 5.20E−13 and buried interface area of 2,544 Å2, which are higher than those observed in the complex with ANPEP. These values indicate that ENPEP might have a higher affinity to RBD than ANPEP. However, the rigid body assumption by these computational docking programs will clearly introduce limitations on accuracy and reliability (86). In particular, there are a limited number of known homologous protein–protein interactions of ENPEP or ANPEP with other viral spike proteins. Therefore, further experiments are needed to verify whether ANPEP or ENPEP can truly bind to SARS-CoV-2 RBD.

Furthermore, as immune responses are critical to SARS-CoV-2 infection, immunological aspects mediated by ANPEP and ENPEP cannot be overlooked. Our analysis revealed that ANPEP and ENPEP expression is highly associated with the immune infiltration of macrophages, monocytes, DCs and NK cells (**Figure 4**), suggesting that ANPEP and ENPEP can play an important role in cellular immunity by regulating the immune infiltrate during GBM-affected by SARS-CoV-2. In fact, increased ANPEP expression is a hallmark of inflammation in neurodegenerative disease, and impaired ANPEP activity has been investigated as a target for anti-inflammatory therapy (87, 88).

**Figure 7:**
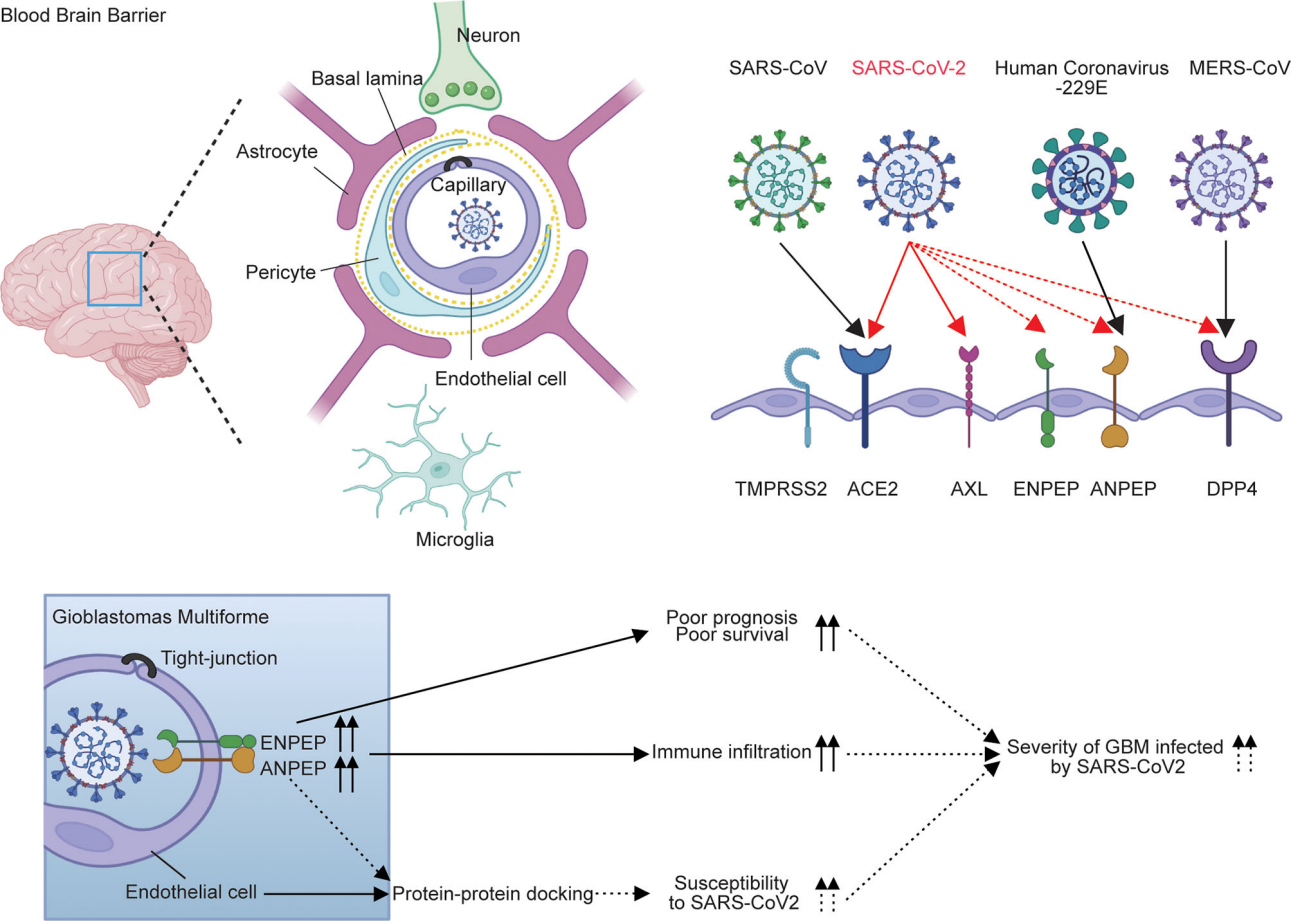
Model for the regulation of ANPEP and ENPEP in GBM against coronavirus infections. Previous studies describe ACE2 and TMPRSS2 as the receptor or co-receptor for SARS-CoV and SARS-CoV-2; AXL for SARS-CoV-2; DPP4 for MERS-CoV; ANPEP for human coronavirus-229E; and DPP4, ENPEP and AENPEP as the candidate receptor for SARS-CoV-2. ANPEP and ENPEP are distributed in endothelial cells of the blood–brain barrier, through which coronaviruses enter the CNS. Protein-protein docking analysis of ANPEP or ENPEP to RBD of SARS-CoV-2 combined with the upregulations of ANPEP and ENPEP in GBM may cause the increase of susceptibility of GBM to SARS-CoV-2. The high levels of ANPEP and ENPEP in GBM is associated with poor survival and high immune infiltration. The overlap of increased risk of GBM to SARS-CoV-2, poor survival, and high immune infiltration may result in the severity of patients with GBM infected by SARS-CoV-2.

Overall, the expression pattern and survival analysis of six receptors in GBM demonstrated that the upregulation of ANPEP and ENPEP is associated with poor survival of GBM. The distribution of ANPEP and ENPEP in endothelial cells of the blood–brain barrier provides the place for SARS-CoV-2 cell entry into the brain, and the potential binding of ANPEP or ENPEP to RBD by protein–protein docking offers tools for SARS-CoV-2 infection, which in turn contributes to the increased susceptibility of GBM to SARS-CoV-2 (**Figure 2C** and **Supplementary Figure 3**). Therefore, the overlap of poor survival, increased risk of GBM to SARS-CoV-2, and high immune infiltration might result in the severity of patients with GBM infected by SARS-CoV-2. The possible conclusion is supported by 39% mortality of GBM-SARS-CoV-2 in one cohort in France (**Figure 7**) (16). This study uncovers the relationship between COVID-19 and GBM. We explored the association of coronavirus receptors with GBM and identified ANPEP and ENPEP as potential biomarkers and therapies for COVID-19 and GBM.

## Data Availability Statement

The data that support the findings of this study are available from the corresponding author upon reasonable request.

## Ethics Statement

The studies involving human participants were reviewed and approved by the Shanghai Outdo Biotech Company Ethics Committee. Written informed consent for participation was not required for this study in accordance with the national legislation and the institutional requirements.

## Author Contributions

JY and AC designed the research. AC, WZ, XLL, GS, ZM, LP, ZS, XGL, and JY performed the experiments and analyzed the data for this work. JY and AC wrote the manuscript. All authors listed have made a substantial, direct, and intellectual contribution to the work and approved it for publication.

## Conflict of Interest

The authors declare that the research was conducted in the absence of any commercial or financial relationships that could be construed as a potential conflict of interest.

## Publisher’s Note

All claims expressed in this article are solely those of the authors and do not necessarily represent those of their affiliated organizations, or those of the publisher, the editors and the reviewers. Any product that may be evaluated in this article, or claim that may be made by its manufacturer, is not guaranteed or endorsed by the publisher.

## Supplementary Material

Supplementary Figure 1 Expression pattern of coronavirus receptors in different subgroups of GBM patients according to the UALCAN program. Box plot showing the relative expression of receptors by **(A)** Age: 21-40 years old (n=12), 41-60 years old (n=67), 61-80 years old (n=69), and 81-100 years old (n=7); b Gender: male (n=101) and female (n=54); and **(C)** RACE: Caucasian (n=139), African-American (n=10), and Asian (n=5). GBM, glioblastoma. *, *P*<0.05; **, *P*<0.01; ***, *P*<0.001.

Supplementary Figure 2 Kaplan–Meier survival curves in CGGA datasets and multivariate Cox analysis. **(A)** Kaplan–Meier survival curves. Red indicates high expression, and blue indicates low expression. **(B)** Forest plot for multivariate Cox analysis between coronavirus receptor expression and some clinicopathological variables in the CGGA database (n=216).

Supplementary Figure 3 Docking of SARS-CoV-2 RBD (isolated from 6M0J) against ANPEP (4FYQ) and ENPEP (4KX7) by the ZDOCK algorithm. **(A)** Binding free energy (kcal-mol-1), binding affinity *Kd* and buried interface area of Top 1 prediction of ANPEP and ENPEP in complex with SARS-CoV-2 RBD. **(B)** Docking model of the SARS-CoV-2 RBD with ENPEP. The binding sites of RBD surface are indicated by hot pink, and the binding sites of ENPEP surface are indicated by green (left). Hydrogen bonds at the interface of amino acids from two proteins are represented by green lines (right).
