# Supplementary figures and images for: Comprehensive Oncogenic Features of Coronavirus Receptors in Glioblastoma Multiforme
Source: Front Immunol. 2022 Apr 6;13:840785. doi: 10.3389/fimmu.2022.840785 (PMC9020264; doi:10.3389/fimmu.2022.840785)

**Supplementary Figure 1**

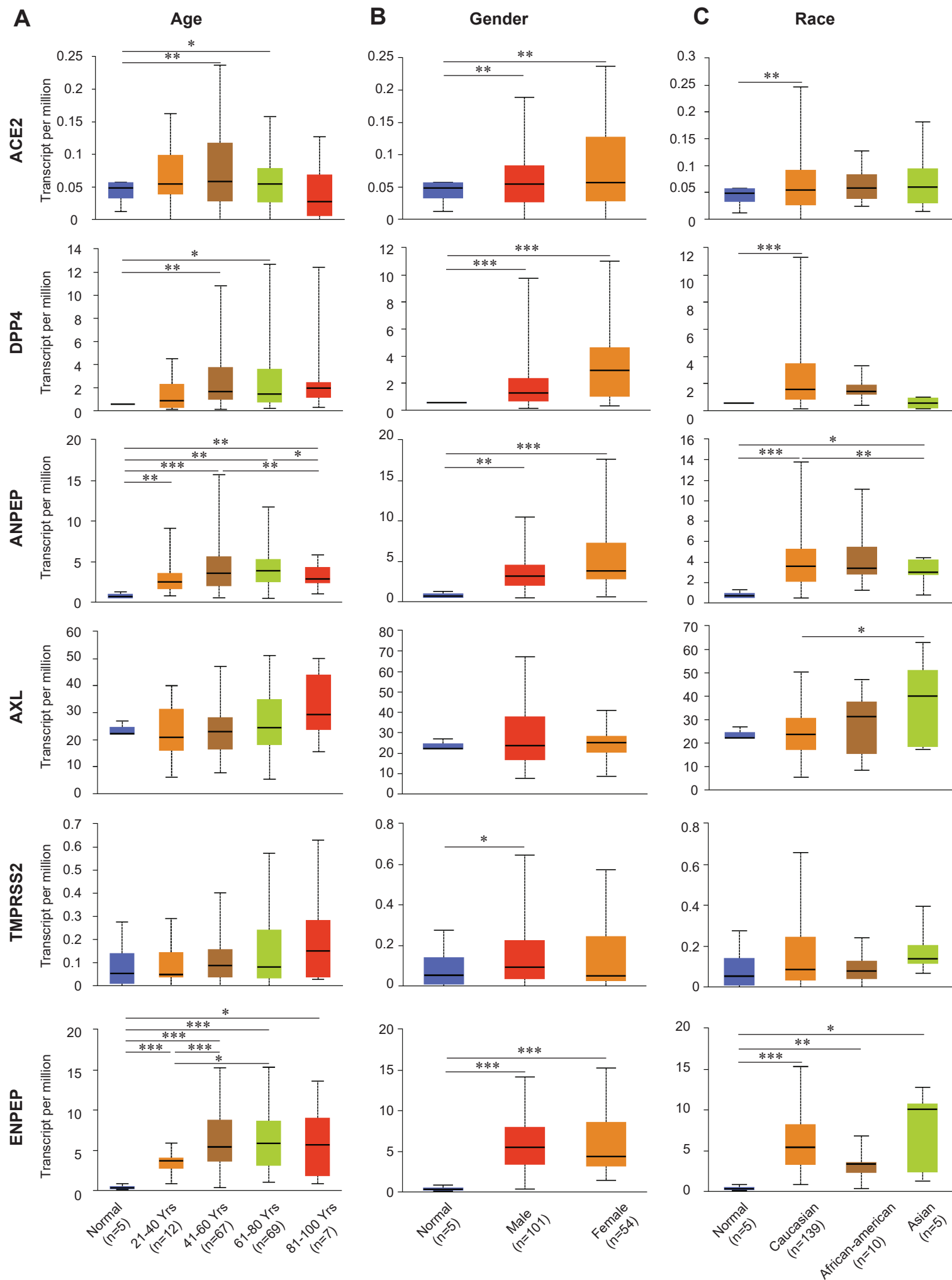

Supplement: Supplementary Figure 1 — Expression pattern of coronavirus receptors in different subgroups of GBM patients according to the UALCAN program. Box plot showing the relative expression of receptors by (A) Age: 21-40 years old (n=12), 41-60 years old (n=67), 61-80 years old (n=69), and 81-100 years old (n=7); b Gender: male (n=101) and female (n=54); and (C) RACE: Caucasian (n=139), African-American (n=10), and Asian (n=5). GBM, glioblastoma. *, P<0.05; **, P<0.01; ***, P<0.001. [file DataSheet_1.pdf]

Supplementary Figure 2

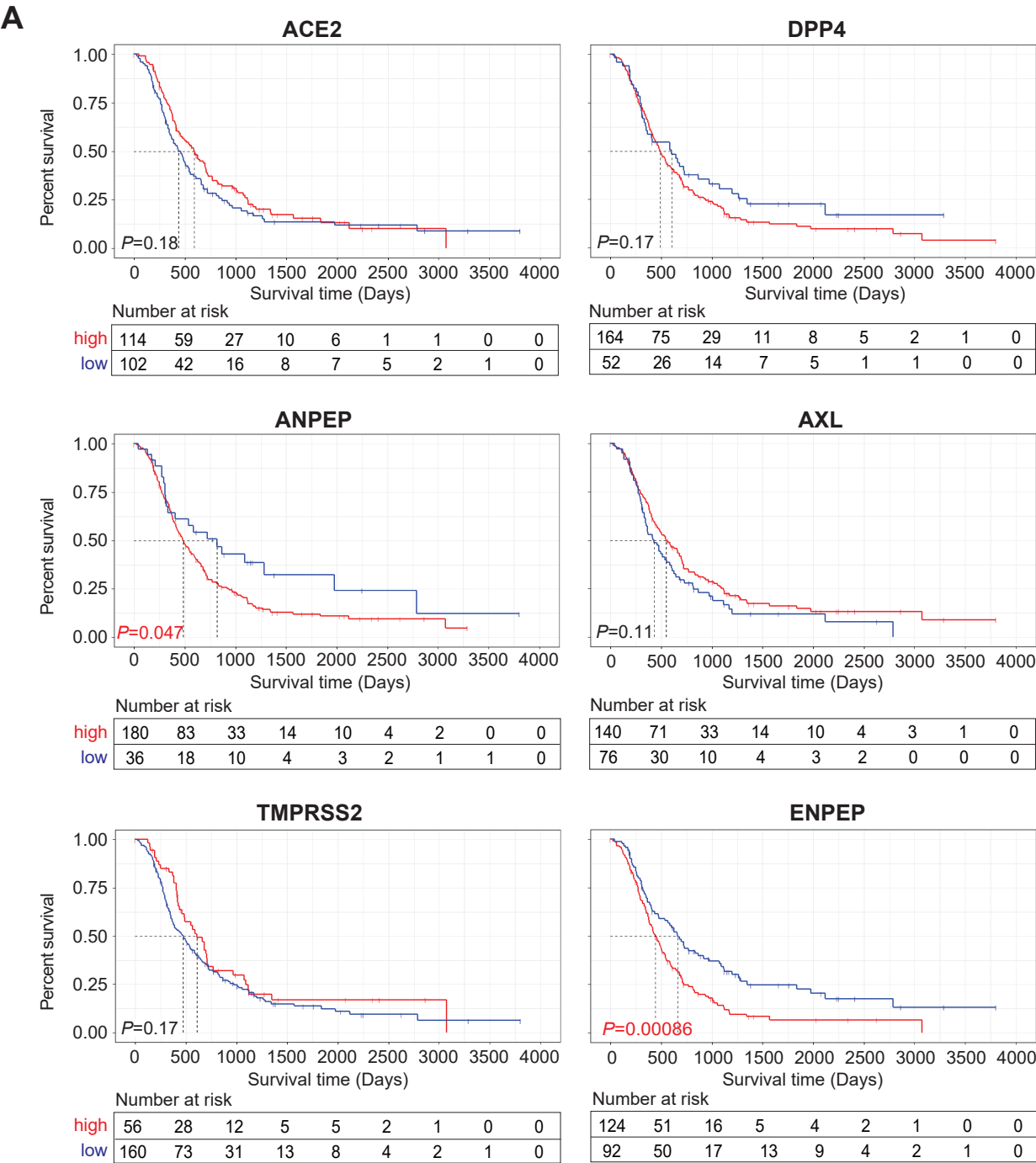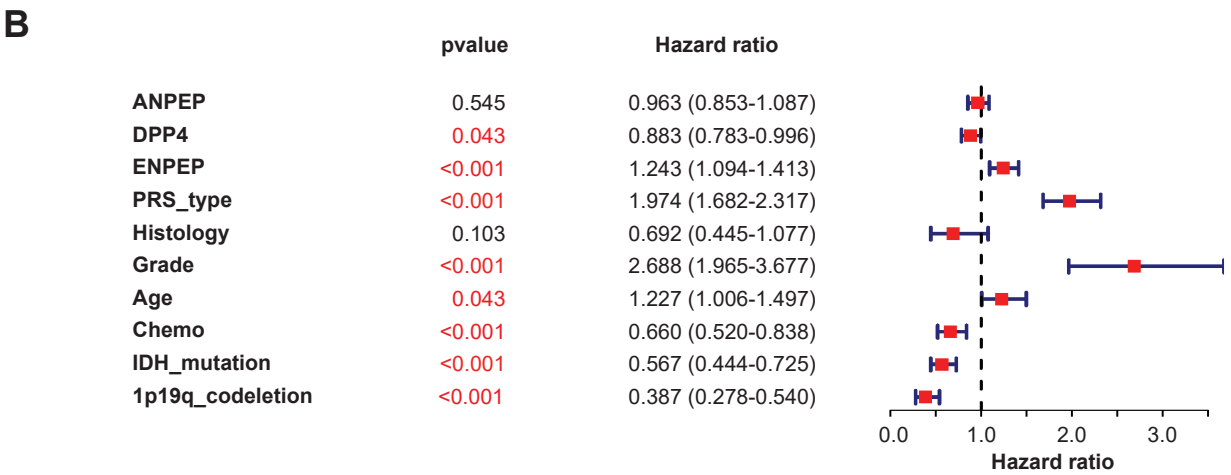

Supplement: Supplementary Figure 2 — Kaplan–Meier survival curves in CGGA datasets and multivariate Cox analysis. (A) Kaplan–Meier survival curves. Red indicates high expression, and blue indicates low expression. (B) Forest plot for multivariate Cox analysis between coronavirus receptor expression and some clinicopathological variables in the CGGA database (n=216). [file DataSheet_2.pdf]
