# Supplementary material for: Comprehensive Oncogenic Features of Coronavirus Receptors in Glioblastoma Multiforme
Source: Front Immunol. 2022 Apr 6;13:840785. doi: 10.3389/fimmu.2022.840785 (PMC9020264; doi:10.3389/fimmu.2022.840785)

Supplementary Figure 3

A

Dock subjects	△G (kcal mol-1)	Kd (M) at 25.0 ℃	Buried surface area (Å2)
RBD-ENPEP	-16.7	5.20E-13	2544
RBD-ANPEP	-14.7	1.60E-11	1962.6

B

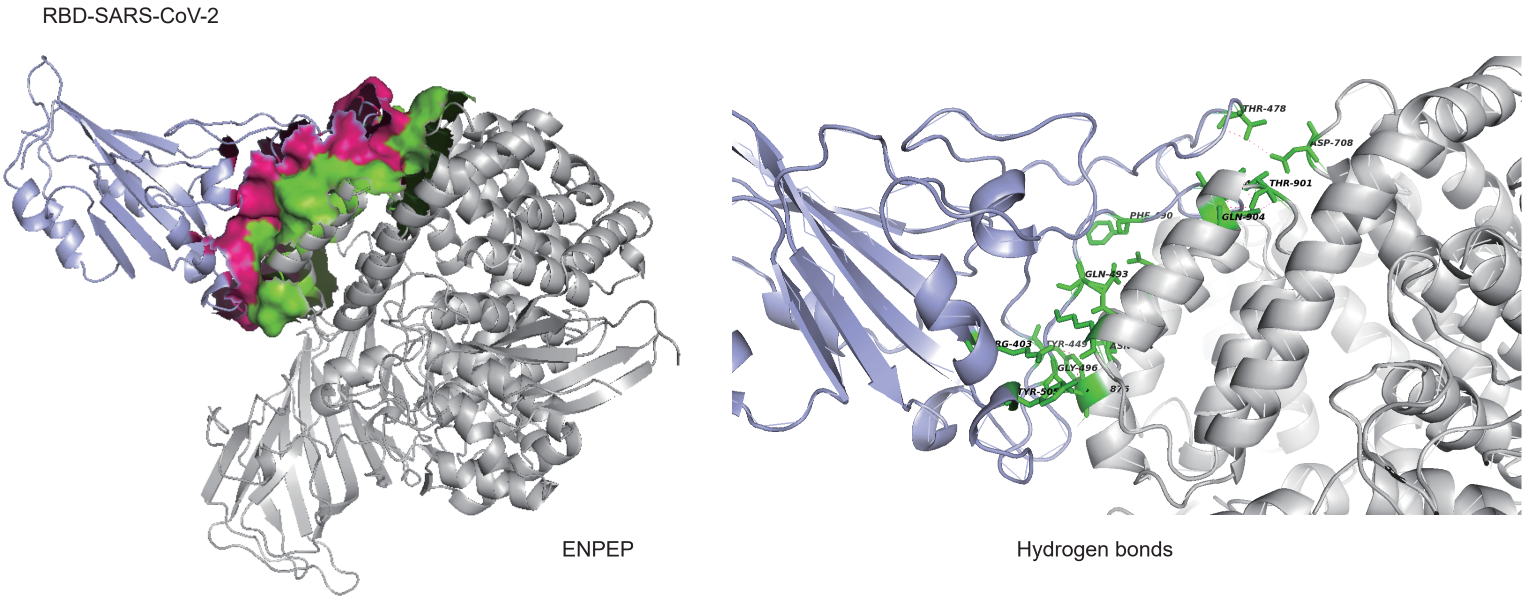

Supplement: Supplementary Figure 3 — Docking of SARS-CoV-2 RBD (isolated from 6M0J) against ANPEP (4FYQ) and ENPEP (4KX7) by the ZDOCK algorithm. (A) Binding free energy (kcal-mol-1), binding affinity Kd and buried interface area of Top 1 prediction of ANPEP and ENPEP in complex with SARS-CoV-2 RBD. (B) Docking model of the SARS-CoV-2 RBD with ENPEP. The binding sites of RBD surface are indicated by hot pink, and the binding sites of ENPEP surface are indicated by green (left). Hydrogen bonds at the interface of amino acids from two proteins are represented by green lines (right). [file DataSheet_3.pdf]
